# Lipids and Lipoproteins in Health and Disease

**DOI:** 10.3390/biomedicines10010087

**Published:** 2021-12-31

**Authors:** Evgeny E. Bezsonov, Igor A. Sobenin, Alexander N. Orekhov

**Affiliations:** 1 Laboratory of Angiopathology, Institute of General Pathology and Pathophysiology, 8 Baltiiskaya Street, 125315 Moscow, Russia; igor.sobenin@gmail.com (I.A.S.); a.h.opexob@gmail.com (A.N.O.); 2 Laboratory of Cellular and Molecular Pathology of Cardiovascular System, Institute of Human Morphology (A. P. Avtsyn Research Institute of Human Morphology), 3 Tsyurupa Street, 117418 Moscow, Russia; 3 Department of Biology and General Genetics, I. M. Sechenov First Moscow State Medical University (Sechenov University), 8 Izmailovsky Boulevard, 105043 Moscow, Russia; 4 National Medical Research Center of Cardiology, Laboratory of Medical Genetics, Institute of Experimental Cardiology, 15a 3rd Cherepkovskaya Street, 121552 Moscow, Russia

This Special Issue, “Lipids and Lipoproteins in Health and Disease: Focus on Targeting Atherosclerosis”, contains research articles and reviews devoted to the study of lipids in different processes, with a focus on the pathological changes that happen during atherosclerosis. It is known that lipid depositions are one of the earliest changes that happen in the intima of arteries upon the above-mentioned disease development. Multiple-modified low-density lipoproteins (LDL) were found to be atherogenic factors circulating in the blood of atherosclerotic patients. These modifications include the existence of desialylated, oxidized, small dense, and electronegative LDL particles. It should be noted that the role of oxidized LDL (oxLDL) in atherosclerosis development is still a debatable subject, starting from the somewhat contradictory information on the association of oxLDL with disease progression, and ending with the absence of noticeable amounts of oxLDL in the blood of most humans. High-density lipoproteins (HDL) participate in the transport of cholesterol from the arterial wall together with many other activities and, thus, play an important function in regulating lipid levels and even inhibiting atherosclerosis. There is a connection between atherogenic LDL, mitochondrial DNA mutations, and chronic inflammation, and the establishment of the details of such a connection will help to solve long-standing atherosclerosis “puzzle”. The current “enigma” of factors related to atherosclerosis development is shown in Figure 1.

This Special Issue presents a variety of research articles and reviews devoted to multiple topics about lipids, lipoproteins, and atherosclerosis, including propositions of new approaches to diagnostics and treatment. All published papers will be mentioned briefly below. 

The details of lipoprotein and lipid modifications related to atherosclerosis, as well as current trends in the pharmacotherapy of this pathology, are reviewed and discussed [1].

The review by Mezentsev and coauthors is devoted to the role of proatherogenic sialidases and desialylated low-density lipoproteins and high-density lipoproteins in atherosclerosis development. Attention is also paid to the immunogenicity of lipoproteins, animal models for atherosclerosis research, and the evaluation of potential therapeutic approaches to treat this disease [2].

Another review focuses on the formation of oxidized low-density lipoproteins, the recognition of oxidation-specific epitopes by macrophages, and the impact of these processes on atherosclerosis development [3].

An attempt was made to connect oxLDL with cancer as a risk factor. In the research paper devoted to the study of oxLDLs and their effects on head and neck cancer cell lines, it was found that the addition of oxLDL increased CD36 expression, leading to an increased lipid uptake and reduced cell migration [4].

The importance of high-density lipoproteins as modulators of immune cells’ activities is described in detail [5].

The effect of obesity on high-density lipoprotein metabolism (composition, distribution, function, activities of lipoprotein-modifying enzymes) was studied in women [6].

Nanoparticles mimicking natural HDL were designed and tested for their ability to induce cholesterol efflux in several cell models. It was found that 1,2-dipalmitoyl-sn-glycero-3-phosphocholine-containing nanoparticles (resembling nascent HDL) induced cholesterol efflux in all models studied, with the increased strength of the effect upon upregulation of the ABCA1 transporter [7].

In another article devoted to the use of HDL-mimicking iron nanoparticles coated with carbon, the authors discovered a high biocompatibility of these particles with human endothelial cell culture and showed their ability to cause structural changes in atherosclerotic plaques ex vivo [8].

The important question related to the characterization of plasma lipoproteins from patients with severe COVID-19 was addressed with the study of particle numbers, sizes, and different metabolites [9].

Several potential biomarkers (miRNAs) of carotid artery stenosis were identified (by the effect on ABCA1) [10].

It was found that the plasma levels of circulating proprotein convertase subtilisin/kexin type 9 (PCSK9), an important protein affecting the metabolism of cholesterol, did not correlate with subclinical atherosclerosis of carotid arteries and damage of vessels in patients without symptoms of cardiovascular diseases [11].

The effect of aging on reverse cholesterol transport and the expression of membrane cholesterol transporters in mice was studied, leading to the conclusion that susceptibility to atherosclerosis with aging must be increased due to the transporters’ dysregulated expression [12].

In a study involving middle-aged adults without cardiovascular disease, it was found that deficiency of vitamin D was associated with high levels of pro-atherogenic, small dense, low-density lipoprotein-cholesterol [13].

The current understanding of the role of vitamin D in atherosclerosis development and lipid metabolism is reviewed [14].

The effects of hepatocyte- and adipocyte-specific knockouts of the atypical chemokine receptor 3 (ACKR3) in ApoE-deficient mice on lipid levels under hyperlipidemic conditions were studied [15]. It was shown that the deletion of ACKR3 in adipose tissues caused a reduction in cholesterol and triglyceride content, but it did not happen in the case of hepatocytes [15].

It was found that there is a pattern of differential expression of miRNAs between a group of metabolically healthy obese and a group of metabolically unhealthy obese individuals, pointing to a connection of miRNAs with metabolic syndrome in case of obesity [16].

Possible new ways for the usage of isomers of hexadecenoic acid (for example, palmitoleic acid) during conditions of inflammation were shown using the model of mouse peritoneal macrophages [17].

Current approaches for reducing lipoprotein(a) levels (one of the cardiovascular risk factors) are discussed in the review, including recent studies on drugs inhibiting the synthesis of lipoprotein(a), based on antisense oligonucleotide and siRNA [18].

In the case of Type 1 Diabetes, pathogenic changes related to dyslipidaemia, atherosclerosis, and atherogenic LDL modifications, as well as modern clinical approaches to manage patients in pediatric and adult populations, are reviewed [19].

The role of lipoprotein lipase in the regulation of atherosclerosis is reviewed in terms of the involvement of this enzyme with some additional factors into intravascular lipolysis, removal of remnant lipoproteins from blood plasma, and promotion of lipoprotein retention in capillaries [20].

The current results of the involvement of scavenger receptor B-class type 1 in the metabolism of different lipoproteins and cardiovascular disease development in mice and humans are discussed [21].

Multiple receptors of fatty acids, including GPR120 (FFAR4), are discussed, including the potential benefits of FFAR4 stimulation as a new strategy to reduce inflammation during atherosclerosis [22].

The current ultrasound methods for visualization and risk evaluation of atherosclerotic plaques are reviewed in detail [23].

The involvement of mitochondria in the process of inflammation during atherosclerosis is discussed [24].

We hope that the next Special Issue will continue to carry the same high standard of publications as this one has.

## Figures and Tables

**Figure 1:**
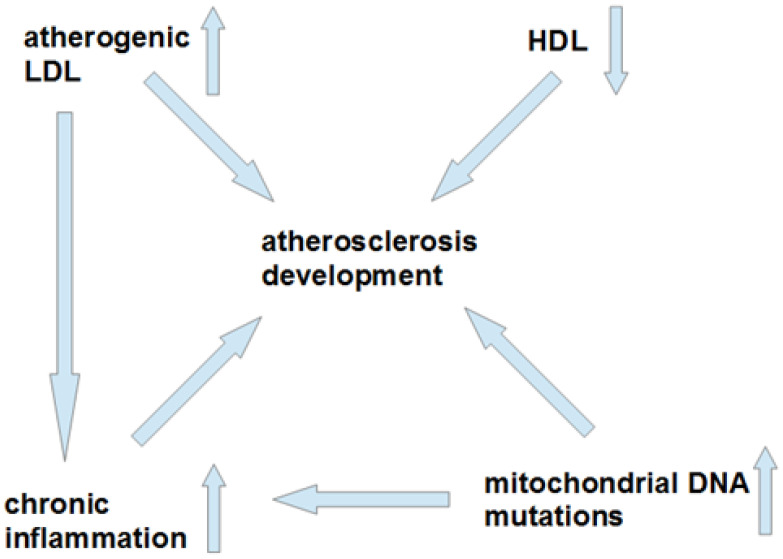
Factors associated with atherosclerosis development and their connections with each other. Chronic inflammation is connected with atherogenic LDL and mitochondrial DNA mutations and, thus, it could be that inflammation is a central component in atherosclerosis pathogenicity. However, we must mention that the exact details of interactions of all these factors with each other (especially, HDL) are still to be clarified in the future. LDL—low-density lipoproteins, HDL—high-density lipoproteins.
